# Trends in obesity, overweight, and malnutrition among children and adolescents in Shenyang, China in 2010 and 2014: a multiple cross-sectional study

**DOI:** 10.1186/s12889-017-4072-7

**Published:** 2017-02-02

**Authors:** Lingling Zhai, Youdan Dong, Yinglong Bai, Wei Wei, Lihong Jia

**Affiliations:** 1 Department of Maternal and Child Health, School of Public Health, China Medical University, No. 77 Puhe Road, Shenyang North New Area, Shenyang, Liaoning Province 110122 People’s Republic of China; 2 Department of Rheumatology, Shengjing Hospital of China Medical University, Shenyang Liaoning Province, People’s Republic of China

**Keywords:** Children, Adolescent, Obesity, Overweight, Malnutrition

## Abstract

**Background:**

The prevalence, characteristics, and trends in obesity, overweight, and malnutrition among children and adolescents in 2010 and 2014 in Shenyang, China was described.

**Methods:**

This was a multiple cross-sectional study using data from the 2010 and 2014 National Survey on Students’ Constitution and Health. A total of 31,031 children and adolescents were included in this survey. Differences in the percentages of obesity, overweight, and malnutrition by age, gender, and living region in 2010 and 2014 were compared using the *χ*2 test. Stepwise logistic regression was performed to select potential covariates for the dependent variable (overweight, obesity, or malnutrition).

**Results:**

The prevalence of obesity and overweight in 2010 was 8.99% and 13.72%, respectively, and 12.64% and 14.06% in 2014, respectively. The prevalence of malnutrition was 10.68% and 10.69% in 2010, and 2014, respectively. In 2010 and 2014, boys and girls 7–11 years of age had higher rates of obesity than other age groups (*P* < 0.01). The prevalence of obesity and overweight was significantly higher in the urban residents compared to the rural residents, and was also significantly higher in boys than girls (*P* < 0.01); however, the prevalence of malnutrition was significantly lower in boys than girls (*P* < 0.01). Compared to 2010, the prevalence of obesity in 2014 increased significantly in boys and girls, and urban and rural residents (*P* < 0.05), but the prevalence of malnutrition did not change. The prevalence of obesity, overweight, and malnutrition was associated with gender, age, and living region by univariate logistic regressions.

**Conclusion:**

The prevalence of obesity and overweight has continuously risen since 2010, and there is a low-age trend of obesity and overweight among children and adolescents in Shenyang, China. The increasing rate of obesity and overweight was faster in rural than urban areas. Malnutrition did not significantly decrease during the 4-year period from 2010–2014.

## Background

Childhood and puberty are the most important periods in our life [1]. A number of diseases acquired during this period may carry over to adulthood or are risk factors for adult diseases [2–6]. Obesity and overweight are not only serious health problems that affect growth and development of children and adolescents, but can also cause developmental problems, such as psychological disorders [7], cognitive dysfunction [8], and influence the timing of puberty [9], and may be accompanied by metabolic syndrome [10]. Malnutrition has always been a concern because malnutrition during childhood may induce health problems in much the same way as obesity [11]; specifically, protein-energy malnutrition and anemia during childhood can delay physical and brain development [12, 13]. There are usually three measures which are used for detecting malnutrition in children: stunting (extremely low height for age); underweight (extremely low weight for age); and wasting (extremely low weight for height). In China, the most common causes of malnutrition among children are inadequate food intake, such as unhealthy dietary habits in urban students, and lack of nutrient-rich food due to poor economic development in rural students [14]. There is a decreasing trend in childhood malnutrition (including anemia and underweight), and the difference in malnutrition between urban and rural students has also decreased with the improvement in living conditions in the past 25 years in China, according to the data of the National Survey on Students’ Constitution and Health (CNSSCH) [15].

A high prevalence of childhood obesity had been reported in many countries [16]. The epidemiologic characteristics of childhood obesity has changed over time in different countries. In the USA, the rate of obesity and overweight increased during the 30 years from 1974–2004 from 5.2% and 10.2% to 17.1% and 16.5%, respectively, and the prevalence of obesity and overweight in 2012 was 17% and 15.0% among children 2–19 years of age, respectively, which did not continue to increase from 2005–2012 [17]. In China, the 2012 National Survey reported that the prevalence of obesity and overweight was 6.4% and 9.6% among children 6–17 years of age, respectively, having increased by 204.8% and 113.3%, respectively, since 2002. The prevalence of stunting and wasting was 3.2% and 9.0% among these children, respectively, having decreased by 49.2% and 32.8%, respectively, since 2002 [18]. The WHO reports that the rate of childhood obesity will continue to increase in developing countries in the future. Thus, the health of children, especially obesity, should be a major focus of attention in China [19].

Shenyang is located in northern China and Liaoning is the provincial capital city; the geography is special and the climate is characterized by a winter > 6 months in duration (the coldest temperature is approximately –28 °C). Southern cities, such as the province of Guangdong and Fujian have approximately 2 months of winter with the coldest temperature of 0 °C. It was well-known that more fat is stored to resist the cold temperatures in the winter, and individuals usually decrease movement in the winter. No studies have reported the prevalence and characteristics of obesity and overweight in Shenyang recently with a large sample. Thus, we described the characteristics of obesity, overweight, and malnutrition among children and adolescents based on 2010 and 2014 data from the CNSSCH from Shenyang, and provide a reference basis for nutritional intervention in children.

## Methods

### Study design and data source

The data were acquired from two cycles in Shenyang of the CNSSCH, which was approved from the working group of the CNSSCH in Shenyang. The sampling methods and physical examinations were the same in all CNSSCH according to the protocols used for previous surveys [20, 21]. Thus, the data from the 2010 and 2014 CNSSCH in Shenyang were comparable and reliable according to two indicators: (1) all the participants were uniformly measured in the same year using the same methods and in the same way; and (2) all participants were school students 7–18 years of age who were selected with stratified random cluster sampling from Shenyang, according to the *Handbook of National Student Physical Health Survey* in 2010, excluding those students who were diagnosed with abnormal growth and development or physical abnormalities. The participants were classified by gender and living region (urban or rural), and each of the four groups had an approximately equal sample size from three socioeconomic classes (upper, middle, and lower). Then, three urban and three rural residential areas were selected. The sample size of each survey was 15,568 and 15,463 in 2010 and 2014, respectively.

In this analysis, children and adolescents were classified into 3 age groups (7–11, 12–14, and 15–18 years), which is a common age range used in the educational system for primary, middle, and high school students in Shenyang, China, respectively.

### Measures

A basic physical examination (height and weight) in schools is required according to the protocol standard of measure for height and weight [22]. All measurements at the survey site were conducted by a team of professionals who had passed a training course in anthropometric measurements.

Height (cm) and weight (kg) were measured using similar instruments at all survey sites [22]. Height was measured to the nearest 0.1 cm using a stadiometer (TZCS-4, Co., Ltd. Xinman Science and Education Equipment, Shanghai, China) while wearing light clothing without shoes and standing erect. Weight was measured to the nearest 0.1 kg using a leveraged scale (RGT-140, Co., Ltd. Xinman Science and Education Equipment) while wearing lightweight clothing without shoes.

BMI was calculated as the body weight (kg) divided by height (m) squared (kg/m^2^). The use of BMI (kg/m^2^) is considered to be a practical and reasonable tool to assess adiposity. Obesity and overweight were defined using the Working Group of Obesity in China [23]. Malnutrition was defined by screening standards for malnutrition of Chinese school-age children and adolescents (WS/T 456-2014). The prevalence of malnutrition was equal to the prevalence of stunting and wasting. Stunting and wasting was defined by using national standards in Chinese school-age children and adolescents (WS/T 456-2014).

### Statistical analyses

Differences in the percentage of obesity, overweight, and malnutrition among boys and girls in 2010 and 2014 were compared using a *χ*2 test. Stepwise logistic regression was performed to select potential covariates for the dependent variable (obesity, overweight, or malnutrition). Normal weight children were selected as a reference for the dependent variable. The independent three variables (age group, gender, and urban vs. rural residence) were entered into logistic regression separately; the adjustment OR for one variable was counted and the other two variables were adjusted. A *P* <0.05 was set to determine the statistical significance. All analyses were conducted with SAS (version 9.2; SAS Institute, Cary, NC, USA).

### Declaration

Ethics approval was granted by the China Medical University, and the study was conducted in accordance with the ethics standards of the Committee on Human Experimentation. The consent procedure was approved by the Ethics Committee of the China Medical University.

## Results

### Characteristics of the study population

As shown in Table 1, the study sample was recruited from primary, middle, and high schools in Shenyang, China. A total of 31,031 children and adolescents were included in the final analysis. In 2010, 7795 children (50.1%) were boys and 7773 children (49.9%) were girls. In 2014, 7740 children (50.1%) were boys and 7723 children (49.9%) were girls. No significant difference existed in the distribution of number by gender, age group, and living region in 2010 and 2014.

**Table 1:** Characteristics of study population in 2010 and 2014

	Age (Year)			Living region		Total
	7–11	12–14	15–18	Urban	Rural	
2010						
Boys	3250	1950	2595	4195	3600	7795
Girls	3250	1950	2573	4173	3600	7773
Total	6500	3900	5168	8368	7200	15568
2014						
Boys	3250	1950	2540	4140	3600	7740
Girls	3249	1950	2524	4123	3600	7723
Total	6499	3900	5064	8263	7200	15463

### Prevalence of obesity, overweight, and malnutrition in 2010 and 2014

The prevalence of obesity and overweight was 8.99% and 13.72% in 2010, respectively, and 12.64% and 14.06% in 2014, respectively. The prevalence of malnutrition was 10.68% and 10.69% in 2010 and 2014, respectively.

As shown in Table 2, both boys and girls in the 7–11 year old group had a much higher rate of obesity than the other age groups in 2010 (*P* < 0.01). The prevalence of obesity decreased in both boys and girls with age. Compared to children who lived in a rural area, the prevalence of obesity and overweight was higher in children who lived in an urban area (*P* < 0.01). The prevalence of obesity and overweight in boys was significantly higher than girls (*P* < 0.01); however, the prevalence of malnutrition in boys was significantly lower than girls (*P* < 0.01).

**Table 2:** The prevalence of obesity, overweight and malnutrition (2010 vs 2014, %)

	Age (Year)			Living region		Total
	7–11	12–14	15–18	Urban	Rural	
2010 Boys						
Obesity	15.72^**^^^	9.13^**^^^	6.78**	13.87^**&&^	7.86^**^	11.10^**^
Overweight	16.95^**^^^	18.00^**^^^	13.95**	17.85^**&&^	14.31^**^	16.22^**^
Malnutrition	8.15^**^^^	9.08	10.48^*^	8.46^**^	9.97^**^	9.16^**^
Girls						
Obesity	10.31^^^^	6.62^^^^	2.76	9.18^&&^	4.22	6.88
Overweight	10.58	12.21	11.23	12.05^&&^	10.22	11.21
Malnutrition	13.42	9.90^^^^	12.40	12.20	12.19	12.20
2014 Boys						
Obesity	18.74^**##^^^	14.10**^##^^^	11.46^**##^	16.81^**&&##^	13.31^**##^	15.18^**##^
Overweight	17.32^**^^^	18.51^**^^^	14.96**	18.09^**&&^	15.42^**^	16.85^**^
Malnutrition	7.97^**^^^	8.31^**^^^	11.38	8.55^**^	9.89^**^	9.17^**^
Girls						
Obesity	13.33^##^^^	9.49^##^^^	5.31^##^	10.62^&&#^	8.72^##^	9.74^#^
Overweight	11.08	11.03	11.69	10.89	11.69	11.27
Malnutrition	13.33^^^	10.77	11.93	11.98	12.50	12.22

vs girls ^*^ *P*<0.05 ^**^ *P* < 0.01; vs rural ^&&^ *P* < 0.01; vs 2010 ^#^ *P* <0.05 ^##^ *P* <0.01; vs 15–19 age group ^^^ *P* <0.05 ^^^^ *P* <0.01

In 2014, both boys and girls in the 7–11 year old group had the highest obesity rates among the three age groups (*P* < 0.01). The prevalence of obesity declined in both boys and girls with age. Compared to children who lived in a rural area, the prevalence of obesity and overweight was higher in children who lived in an urban area (*P* < 0.01); the prevalence of malnutrition was lower in children who lived in an urban area. The prevalence of obesity and overweight in boys was significantly higher than girls (*P* < 0.01). The prevalence of malnutrition in boys was significantly lower than girls (*P* < 0.01; Table 2).

Compared to 2010, the prevalence of obesity increased significantly in boys and girls who lived in urban and rural areas (*P* < 0.05). The prevalence of malnutrition did not change significantly in 2014 (Table 2).

### Velocity of the rate increase in obesity, overweight, and malnutrition between 2010 and 2014

As shown in Table 3, the prevalence of obesity increased each year among all of the age groups. The increase in velocity of obesity in the 15–18 year old group was highest, followed by the 12–14 year old group; the velocity of obesity in the 7–11 year old group was the lowest.

**Table 3:** The velocity of rate increase of obesity, overweight and malnutrition (2010–2014, %)

	Age (Year)			Living region		Average
	7–11	12–14	15–18	Urban	Rural	
Boys						
Obesity	19.2	54.4	69.0*	21.2	69.3^**^	36.76
Overweight	2.2	2.8	7.2	1.6	7.8^**^	3.9
Malnutrition	-2.2	-8.5	8.6	1.1	-0.8	0.1
Girls						
Obesity	29.3	43.4	92.4*	15.7	106.7^**^	41.6
Overweight	11.5	-9.7	4.1	-9.6	14.4^**^	0.5
Malnutrition	-0.7	8.8	-3.8	-1.8	2.54	0.2

vs 7–12 age group ^*^ *P* < 0.05; vs urban ^**^ *P* < 0.05

As shown in Table 3, the prevalence of obesity increased each year in children who lived in urban and rural areas; however, the increased velocity of obesity in the rural areas was higher than the urban areas. There was no apparent change in the increased velocity with respect to overweight and malnourished children.

### Multivariate logistic regression model predicting obesity, overweight, and malnutrition

The results of univariate logistic regression on obesity, overweight, and malnutrition are shown in Table 4. Analysis of univariate logistic regression indicated that obesity, overweight, and malnutrition are associated with gender, age group, and living region. These variables were used as covariates in the multivariate logistic regression model.

**Table 4:** Summary of multivariate logistic regression model predicting obesity, overweight and malnutrition

Variables	Obesity	Overweight	Malnutrition
	OR^a^(95%CI)	OR^a^(95%CI)	OR^a^(95%CI)
Age group(year)			
7–11	2.52(2.29–2.77)	1.22 (1.13–1.32)	1.05(0.97–1.14)
12-14	1.58(1.41–1.76)	1.21 (1.11–1.32)	0.86(0.78–0.95)
15–18(reference)			
Living region			
Urban(reference)			
Rural	0.62(0.58–0.67)	0.81(0.76–0.87)	1.03(0.96–1.11)
Gender			
Boys(reference)			
Girls	0.56(0.52-0.60)	0.61(0.57-0.65)	1.30(1.20-1.39)

^a^Adjustment OR

As shown in Table 4, the students attending primary and middle schools were more likely to be obese than high school students (adjusted odds ratio [aOR]: 2.52, 95% CI: 1.53–3.58 and aOR: 1.58, 95% CI: 1.41–1.76, respectively). Children and adolescents in the 7–11 and 12–14 year old groups were approximately 1.2 times (95% CI: 1.13–1.32 and 1.11–1.32, respectively) more likely to be overweight than adolescents in the 15–18 year age group. The aOR for malnutrition in children and adolescents in the 12–14 year old group was 0.86 (95% CI: 0.78–0.95) compared to adolescents in the 15–18 year old group. This finding suggested that high school students are more likely to be malnourished than middle school students. Thus, age may be a critical factor in predicting obesity, overweight, and malnutrition among children and adolescents.

As shown in Table 4, the aOR for gender with obesity was 0.56 (95% CI: 0.52–0.60). This finding suggested that girls were less likely to be obese than boys. Similarly, girls were less likely to be overweight than boys (aOR = 0.61, 95% CI: 0.57–0.65); however, girls were 1.30 times (95% CI: 1.20–1.39) more likely to be malnourished than boys. This finding suggested that girls are more likely to be malnourished than boys.

The aOR for obese children who live in urban and rural areas was 0.62 (95% CI: 0.58–0.67). This finding suggested that rural residence was less likely to be associated with obesity than urban residence. Similarly, rural residence was less likely to be associated with overweight than urban residence (aOR = 0.81, 95% CI: 0.76–0.87; Table 4).

## Discussion

The prevalence of obesity and overweight in 2010 was 8.99% and13.72%, respectively, and 12.64% and 14.06% in 2014, respectively. The rate of obesity was significantly higher in 2014 than 2010; the average increased rate of obesity reached 40.60% during the 4 years. The rate of overweight was not statistically different between 2010 and 2014. The prevalence of obesity and overweight was 6.4% and 9.6% in 2012 for Chinese school-aged children, respectively [24]. The rate of obesity in Shenyang school-aged children in 2010 surpassed the national average in 2012. In 2014, 26.5% of children and adolescents were obese or overweight. Recent estimates suggest that approximately 32% of US children and adolescents 2–19 years of age were obese or overweight in 2009–2010 [25]. In Europe, the prevalence of obesity ranged from 6.0%–26.6% among boys and 4.6%–17.3% among girls 6–9 years of age in 12 countries, according to 2008 data from the WHO [26]. The prevalence of overweight or obesity in Shenyang in 2014 was similar to the US and Europe.

In the current study, we also found a relatively high rate of malnutrition (approximately 9% in boys and 12% in girls) among the children and adolescents in Shenyang from 2010–2014, with no statistically significant difference during the 4 years. The results of multiple analysis showed that age, living region, and gender are risk factors for obesity, overweight, and malnutrition.

### Age

The prevalence of obesity and overweight was highest for boys and girls in the 7–11 year old group among all age groups in 2010 and 2014. Logistical analysis results showed that the 7–11 year old group was 2.52 times (95% CI: 2.29–2.77) more likely to be obese than the 15–18 year old group, and the 12–14 year old group was 1.58 times (95% CI:1.41–1.76) more likely to be obese than the 15–18 year old group. Indeed, younger boys and girls were more like to be obese. The younger the age of onset of obesity, the higher the prevalence of obesity-related disease, such as fatty liver disease, obstructive sleep apnea, type 2 diabetes, and a variety of orthopedic problems [27]. Thus, preventing obesity should begin in younger children [28]. With economic growth, there has been an accompanying low-age trend for obesity and overweight with the earlier introduction of fast food, watching television, and playing computer games. Moreover, the attitude of elders regarding body weight with well-being during childhood might play an important role in the increasing prevalence of low-age trend of obesity.

The velocity of the rate increase of obesity and overweight in older children was more rapid than younger children in our study. This finding may be was associated with puberty for children after 12 years of age, as well as the increase in sedentary and decrease in activity times, in part because of the emphasis on learning with age. Thus, obesity prevention measures should be implemented in all age groups.

### Living region

There was a significantly higher prevalence of obesity and overweight in urban students than rural students, which was consistent with the results of other studies conducted in China [22, 29]. The difference in socioeconomic status (SES) between urban and rural areas in China has been shown to influence the prevalence of childhood obesity and overweight [22]. The difference in dietary patterns between urban and rural residents in China might also explain these findings [30]. In China, urban residents have a higher SES than rural residents. National surveys have demonstrated a higher consumption of energy-dense foods, such as animal-based foods, and a lower consumption of vegetables and fruits amongst urban children compared with rural children [31, 32]. Compared with rural families, urban families own more electronic products, such as video players and computers. In addition, more children in urban areas are transported to school by automobiles, instead of bicycling or walking, which is probably a contributing factor to childhood obesity [30, 32].

The present study showed that the increasing rate of obesity in rural students was more rapid than urban students during 4 years beginning in 2010 (82.3% vs. 19% and 10.6% vs -3.1%, respectively). From 2011, a nutrition improvement program in rural areas of the compulsory education student was implemented in China. The children's nutrition status improved in rural areas; however, with the improvement in nutrition, the prevalence of obesity and overweight also increased. A rational diet and more physical activity should be advised for all children, especially rural school-aged children [33].

The prevalence of malnutrition was not significantly different in rural children and adolescents compared with urban children and adolescents. The prevalence of malnutrition did not change significantly from 2010–2014 and the prevalence of malnutrition did not increase substantially in rural students, thus the nutrition improvement program in rural areas of the compulsory education student are effective. The difference in nutrition status between urban and rural students was negligible, thus childhood overnutrition and malnutrition should be prevented at the same time.

### Gender

Boys had a higher prevalence of obesity and overweight than girls, and the prevalence in urban boys was highest, which is consistent with the results of other studies in China [22, 34]. Logistical analysis results showed that boys are more susceptible to obesity than girls. Studies conducted in Western countries have revealed that there is a gender difference in the prevalence of obesity and overweight among children and adolescents [35, 36]. Indeed, boys and girls differ in genetic and environmental factors (ethnicity, hormone biology, body composition, patterns of weight gain, and susceptibility to social factors), which may lead to these gender differences [30, 37]. Moreover, there exists a traditional, societal preference for boys, especially in China where boys are likely to enjoy more of the family’s resources than girls [30, 38]. Although boys showed a higher prevalence of obesity and overweight than girls, the velocity of the rate increase of obesity in girls was also high (41.6%), especially in the 15–18 year old group. Clearly, more attention should be paid to obesity in boys and girls.

Girls showed a higher prevalence of malnutrition than boys, which is consistent with the results of other studies in China [22, 39]. The basis for this finding may be that girls are likely to enjoy less of the family’s resources, pubertal girls pay more attention to their physical appearance, a malignant diet was followed in some non-obese, non-overweight girls, and the social pressure preferring thinness among girls [40].

### Limitations

The current study analyzed the prevalence of obesity, overweight, and malnutrition by age, gender, and living region from cross-sectional representative surveys in Shenyang, but did not analyze the risk factors for obesity, overweight, and malnutrition. The current study showed the epidemiologic characteristics of obesity, overweight, and malnutrition in school-aged children in Shenyang, which is the latest data available. It is beneficial for us to adopt targeted measures to prevent childhood obesity and malnutrition.

## Conclusions

In conclusion, the prevalence of obesity and overweight among children and adolescents has continuously risen each year since 2010 in Shenyang, and is above the national levels. The increased rate of obesity was more rapid amongst rural areas than urban areas, and showed a low-age trend of obesity and overweight. The prevalence of malnutrition did not significantly decrease during the 4-year study period. The prevalence of obesity, overweight, and malnutrition was shown to be associated with gender, age, and living region.

## Abbreviation

CNSSCH: National Survey on Students’ Constitution and Health
